# IOLs for cataract surgery

**Published:** 2025-09-10

**Authors:** Suganya Anbalagan, Aravind Haripriya, Ravilla D Ravindran

**Affiliations:** 1 Medical Consultant: Cataract and IOL Services, Aravind Eye Hospital, India.; 2 Chief: Cataract and IOL services, Aravind Eye Hospital, India.; 3 Chairman: Aravind Eye Care System, India.

**Achieving quality refractive outcomes after cataract surgery depends not only on surgical skill, but also on thoughtful IOL selection and strong logistical planning.**

**Figure:**
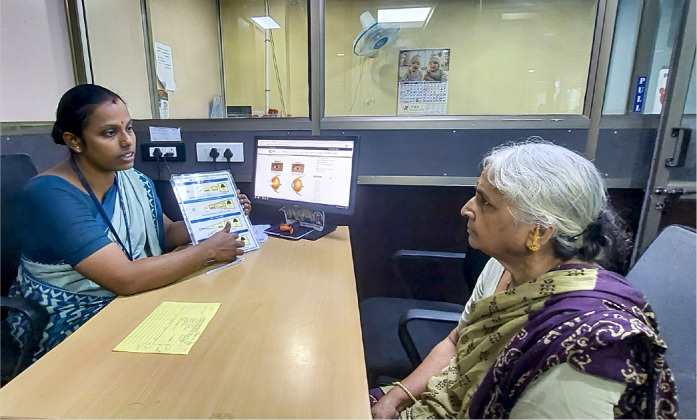
Patient counselling on types of IOLs. INDIA

© ARAVIND EYE CARE SYSTEMS. CC BY-NC-SA 4.0

In low- and/or middle-income countries, manual small-incision cataract surgery (MSICS) using rigid polymethylmethacrylate (PMMA) monofocal intraocular lenses (IOLs) has remained the default option for decades, as MSICS can be performed in basic clinical settings, and because these are the most affordable type of lens, costing around USD 17 in Africa and as little as USD 3–4 for a locally manufactured IOL in India. However, in recent years, an increasing number of IOL types have become available worldwide (see panel). In low-resource settings, the challenge is to balance good clinical practice with patient needs, costs, and infrastructure.

In resource-limited settings, a pragmatic approach, focusing on monofocal and advanced monofocal lenses, selective use of toric lenses, and efficient inventory management, can help to ensure cost effective personalised care with the best possible refractive outcomes.

## Tailoring IOL selection

Clinical factors such as astigmatism, ocular surface health, macular status, and surgical history help guide the selection of an IOL. In patients who have undergone refractive procedures, advanced biometry techniques or the Barrett True-K formula are needed to accurately calculate the lens power needed.^1^

The patient's capacity to pay also significantly influences the IOL choice among self-paying individuals. However, even when they can pay, it is essential to consider the patient's expectations, lifestyle, and personality. Using a questionnaire to assess patient's visual needs and preferences makes the choice of IOL and counselling easier for the provider and patients. For example, extended depth of focus (EDOF) or advanced monofocal lenses (see panel) may be well suited to active, working-age individuals. In contrast, standard monofocal lenses typically meet the needs of older adults or patients with lower visual expectations. Multifocal IOLs offer spectacle independence, but may cause glare, making them less suitable for patients with retinal disease or those who are particular about clarity.^2,3^

The availability of operating infrastructure also affects IOL options. In basic surgical settings, MSICS with rigid PMMA monofocal IOLs remains the default option. In centres with phacoemulsification and advanced diagnostic tools, premium options such as toric and multifocal lenses can be offered. However, in low-resource settings, routine use of multifocal or toric IOLs is often not practical due to the high cost of these IOLs and the lack of infrastructure needed to ensure precision in assessment and surgery.^1,2^

## Inventory management strategies

Inventory management requires good local logistics and communication between the medical and procurement teams. A yearly audit of lenses used can help to predict the numbers needed so they can be ordered in advance (see the KCMC case study in this article), ensuring that you have a baseline stock of both standard and less frequently used dioptres.

Here are some additional strategies:^4^

- Categorise inventory by IOL type, then IOL power
- Use a first-in, first-out (FIFO) system to prevent wastage due to expiry
- Track monthly usage to ensure there is enough stock. Use spreadsheets or tracking software to monitor expiry dates, usage rates, and reorder points
- Order rare IOL powers or advanced IOLs after the patient confirms they would like to go ahead, and schedule the operation once you know when the IOLs will arrive. This avoids the cost associated with holding a costly inventory of different powers and reduces the risk of expiry
- Maintain regular communication with suppliers to enable timely restocking.

As per National Institute for Health and Care Excellence (NICE) guidelines, one matching IOL should be in the operating room, with an identical backup available. For routine cataract surgery, two sets are usually enough. In patients at risk of complications such as posterior capsular rupture, [A1] a backup multipiece IOL of the same power must be available, regardless of the technique used.^4^

**Main types of IOL, by visual outcome**

**1. Monofocal IOLs**

**Figure 1:**
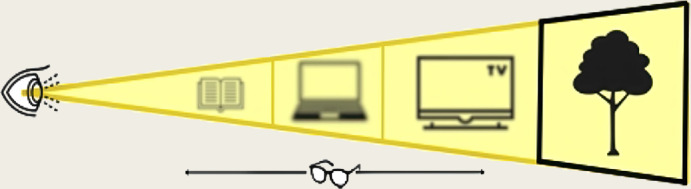
Monofocal IOLs offer clear vision at a single focal point, e.g. at distance (in this instance).

Monofocal IOLs offer clear vision at a single focal point, e.g. at distance (Figure 1). These are used in most cataract operations.

© AECS CC BY-NC-SA 4.0

The most affordable type of monofocal IOL are **polymethylmethacrylate (PMMA)** lenses. They are rigid, not foldable, and are inserted using the manual small-incision cataract surgery (MSICS) technique.

**Hydrophilic** and **hydrophobic acrylic** IOLs also have a single focal point. They are foldable and can therefore be used in phacoemulsification cataract surgery, which creates a much smaller incision compared to MSICS. Hydrophobic lenses are more expensive (USD 40–90), but they are popular for use in children as they give optimal results with the least amount of inflammation.^5^

**Visual outcome.** Monofocal IOLs provide clear vision at a single distance only. The choice of distance depends on the patient and their needs, as described in another article in this issue. Many patients choose to be corrected for distance vision, and then typically require spectacles for near and intermediate tasks. Patients with a history of myopia may choose to have clear intermediate vision instead; this helps them to retain some near vision without relying on spectacles. They would need spectacles for distance vision. Patients can also be offered **monovision**, which means the IOL for one eye provides clear distance vision, and the IOL in the other eye provides clear near vision.

**2. Advanced monofocals**

These lenses have a broader depth of focus and enhanced contrast, thereby improving functional intermediate sight and distance vision. They are ideal for drivers, children, and people who work in low light.^5^ These lenses offer high optical quality, but patients will still need spectacles for near vision.

**Figure 2:**
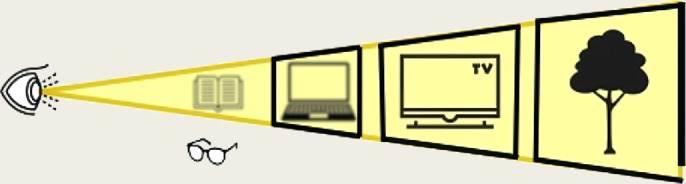
Advanced monofocals offer distance vision with improved intermediate vision.

© AECS CC BY-NC-SA 4.0

**Visual outcome.** Advanced monofocal IOLs offers improved intermediate vision along with distance, reducing dependence on spectacles for mid-range tasks like computer use.

**Limitations.** Advanced monofocals are 4–8 times as expensive as PMMA lenses.

**Multifocal IOLs**

These usually have bifocal or trifocal designs that split light into two or three focal areas (foci) respectively. Trifocal lenses typically distribute light as follows: 50% for distance, 20% for intermediate, and 30% for near.^2^ These lenses offer independence from spectacles at all distances, but they are expensive and not suitable for all patients; their use requires careful patient selection and counselling.^3^

**Figure 3:**
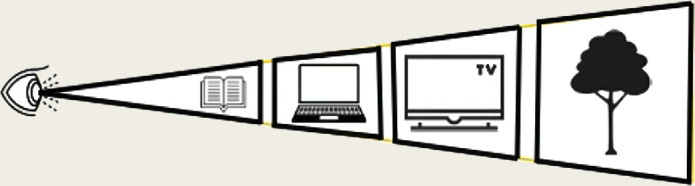
Multifocal offer independence from spectacles at all distances.

© AECS CC BY-NC-SA 4.0

**Visual outcome:** Multifocal/trifocal lenses are designed to offer spectacle-independent vision at different distances (near, intermediate and distant). Up to 90% of patients using these lenses can be spectacle-free.

**Limitations.** Risk of glare, halos, reduced contrast. They are not ideal for patients with retinal disease, ocular pathologies, or glaucoma, as they make it difficult to examine the retina. They are significantly more expensive than PMMA lenses.

**Extended depth-of-focus (EDOF) IOLs**

These extend one focal point across a continuous range rather than splitting light into multiple foci. This helps patients move smoothly between distances, especially for intermediate vision and functional near sight. They cause fewer halos and glare and offer better contrast and night vision compared to multifocal lenses. However, there may still be more glare than with monofocal IOLs. Moreover, some patients may still require spectacles for near work.^6^

**Figure 4:**
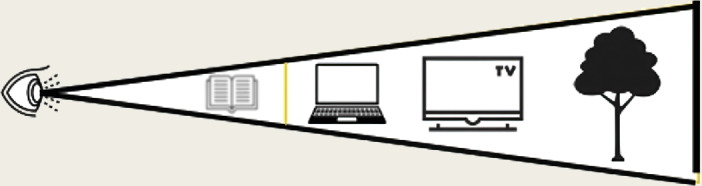
Extended depth-of-focus lenses offer smooth transition between focal distances.

© AECS CC BY-NC-SA 4.0

**Visual outcome.** Extended depth of focus (EDOF) lenses provide a continuous range of vision from intermediate to distance with minimal visual disturbances, and some near vision support.

**Limitations.** Significantly higher cost. May still need near vision spectacles. Some glare still present.

**Toric IOLs**

These are foldable acrylic IOLs that correct regular corneal astigmatism. They give excellent quality of vision when correctly aligned. However, this requires precise preoperative biometry, calculation, planning, and intraoperative placement, as postoperative rotation can compromise their effectiveness.^1^ Toric lenses are available in both monofocal and multifocal types. Monofocal toric IOLs are most commonly used, and more than 90% of these patients achieve Snellen visual acuity of 6/9 or better.
